# A79 THE RELATIONSHIP BETWEEN VISCERAL ADIPOSITY AND NONALCOHOLIC FATTY LIVER DISEASE DIAGNOSED BY CONTROLLED ATTENUATION PARAMETER IN PEOPLE WITH HIV: A PILOT STUDY

**DOI:** 10.1093/jcag/gwac036.079

**Published:** 2023-03-07

**Authors:** W Elgretli, N Paisible, C Costiniuk, J Cox, D Kablawi, M Klein, N Kronfli, J -P Routy, J Falutz, B Lebouche, G Guaraldi, G Sebastiani

**Affiliations:** 1 Division of Experimental Medicine, McGill University; 2 Chronic Viral Illness Service; 3 Division of Gastroenterology and Hepatology, McGill University Health Center; 4 Department of Family Medicine, McGill University, Montreal, Canada; 5 University of Modena and Reggio Emilia; 6 Azienda Ospedaliero-Universitaria di Modena, Modena, Italy

## Abstract

**Background:**

Aging people with HIV (PWH) on antiretroviral therapy face high rates of metabolic dysfunction and nonalcoholic fatty liver disease (NAFLD). Fat alterations are frequent in PWH and predict worse cardiometabolic outcomes. Visceral adipose tissue (VAT) is an important compartment of body fat tissue releasing bioactive molecules. As a hormonally active tissue, VAT critically contributes to obesity-related disorders and is associated with ectopic fat accumulation in the liver.

**Purpose:**

We aimed to investigate NAFLD diagnosed by controlled attenuation parameter (CAP) as a marker of visceral adiposity in PWH.

**Method:**

We conducted a prospective pilot study (ClinicalTrials.gov 2021-6656) of HIV mono-infected patients undergoing metabolic characterization and paired CAP by transient elastography with dual-energy X-ray absorptiometry (DEXA) scan. NAFLD was defined as CAP ≥285 dB/m, in absence of alcohol abuse. Excess visceral adiposity was defined as VAT>1.32 Kg. Pairwise correlation, area under the curve (AUC) and logistic regression analysis were employed to study the association between VAT and CAP.

**Result(s):**

30 patients (90% male, mean age 48.5, mean BMI 29.9, mean waist circumference 100.9, 50% with NAFLD) were included. When compared to those without excess VAT, PWH with excess VAT were older (53+12 vs 43+13 years, p=0.035), had longer duration of HIV infection (20+13 vs. 9+9 years, p=0.021), had higher BMI (32+4 vs 27+4 Kg/m^2^, p=0.002) and waist circumference (107+11 vs. 93+12 cm, p=0.004). They also had more history of cardiovascular events (29% vs. 0, p=0.032) and higher lipid accumulation product, a marker of lipid accumulation based on waist circumference and triglycerides (112+53 vs. 38+27, p<0.001). CAP was higher in PWH with excess VAT (319+52 vs. 213+52 dB/m, p<0.001). CAP positively correlated with all visceral fat measurements by DEXA, including VAT (r=0.650, p<0.001), VAT/body weight ratio (r=0.565, p=0.001) and fat mass (r=0.390, p=0.033). Both BMI and waist circumference showed correlation with VAT and fat mass, but not with VAT/body weight ratio (see Figure). After adjusting for duration of HIV infection (aOR 1.01 per year, 95% CI 0.91-1.12; p=0.921), BMI (aOR 1.77, 95% CI 0.74-4.23; p=0.202) and waist circumference (aOR 0.91 per cm, 95% 0.68-1.21; p=0.509), CAP remained the only independent predictor of excess VAT (aOR 1.05 per dB/m, 95% CI 1.01-1.10; p=0.036). The AUC analysis determined CAP had excellent performance to diagnose excess VAT (AUC 0.92, 95% CI 0.81-1.00), higher than BMI (AUC 0.83, 95% CI 0.68-0.99) and waist circumference (AUC 0.81, 95% CI 0.65-0.97). The optimized CAP cut-off to diagnose excess VAT was 266 dB/m, with a sensitivity of 88.3% and a specificity of 84.6%.

**Image:**

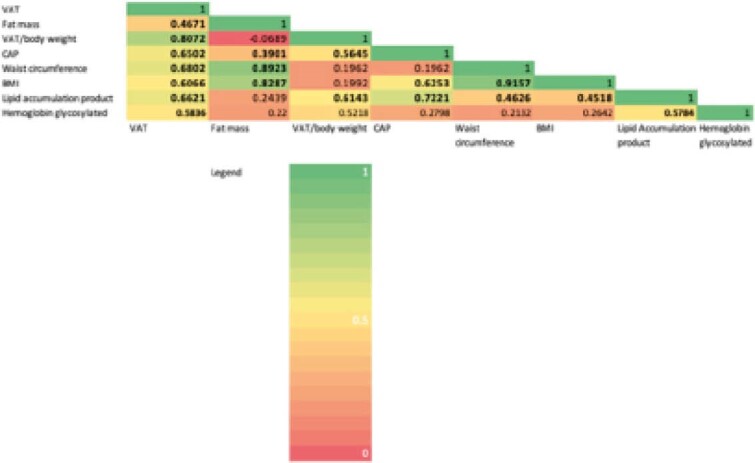

**Conclusion(s):**

NAFLD diagnosed by CAP is associated with VAT in PWH independently of anthropometric measures of obesity. CAP could be used as a diagnostic marker of visceral adiposity in the practice of HIV medicine

**Please acknowledge all funding agencies by checking the applicable boxes below:**

Other

**Please indicate your source of funding;:**

CanHepC

**Disclosure of Interest:**

W. Elgretli Grant / Research support from: CanHepC, N. Paisible: None Declared, C. Costiniuk: None Declared, J. Cox: None Declared, D. Kablawi: None Declared, M. Klein: None Declared, N. Kronfli: None Declared, J.-P. Routy: None Declared, J. Falutz: None Declared, B. Lebouche: None Declared, G. Guaraldi: None Declared, G. Sebastiani: None Declared

